# Empathy and Efficiency in Healthcare at Times of Austerity

**DOI:** 10.1007/s10728-019-00373-x

**Published:** 2019-05-31

**Authors:** Angeliki Kerasidou

**Affiliations:** 1 The Ethox Centre, Nuffield Department of Population Health, University of Oxford, Oxford, England UK; 2 The Wellcome Trust Centre for Ethics and Humanities, Nuffield Department of Population Health, University of Oxford, Oxford, England UK

**Keywords:** Empathy, Efficiency, Austerity, Healthcare, Professionalism

## Abstract

Efficiency is an important value for all publicly funded healthcare systems. Limited resources need to be used prudently and wisely in order to ensure best possible outcomes and waste avoidance. Since 2010, the drive for efficiency, in the UK, has acquired a new impetus, as the country embarked on an ‘age of austerity’ purportedly to balance its books and reduce national deficit. Although the NHS did not suffer any direct budget cuts, the austerity policies imposed on the welfare system, including social and mental healthcare, have had a direct and detrimental impact on the healthcare service. This paper draws from a qualitative study conducted in three A&E Departments in England to explore the effects of austerity policies on the everyday experiences of doctors and nurses working in Emergency Departments. It discusses the operationalisation of efficiency in A&E, in a climate of austerity, and its effects on the experiences and practices of healthcare professionals. It uses the empirical data as a springboard to highlight the role of structures and regulations, in this case targets and protocols, in how core healthcare ethical values, such as empathy, are exercised in practice. It provides an analysis of the normative role structures and regulations can play on the perception and practice of professional duties and obligations in healthcare.

## Introduction

Efficiency is an important value in healthcare provision, especially for publicly funded healthcare systems such as the National Health Service (NHS) in the UK. A population’s health needs will always be greater than the resources available to meet these needs. Limited resources need to be used prudently and wisely in order to ensure best possible outcomes and waste avoidance. In the UK, in the past 8 years the drive for efficiency has acquired a new impetus. In 2010, the UK embarked on an ‘age of austerity’ in an attempt, reportedly, to balance its books and reduce national deficit. The public sector had to reduce expenditure and use existing or even decreasing resources more efficiently [1, 2]. By 2018, the effects of austerity particularly on women, children and the socially disadvantaged were so widespread that the UN Report concluded that the governments since 2010 had inflicted ‘great misery […] unnecessarily’ on the UK citizens [3]. Although the NHS did not suffer any direct budget cuts, the austerity policies imposed on the welfare system, including social and mental health care, have had a direct and detrimental impact on the healthcare service. As the health and care needs of the population keep increasing, the NHS has been struggling to meet the rising demand within the constraints of the available resources. Increasing efficiency, therefore, seemed vital.

This paper draws upon data from a study conducted in three Accident and Emergecy (A&E) Departments in England, which explored the effects of austerity policies on the everyday experiences of doctors and nurses working in Emergency Departments. It discusses the operationalisation of efficiency in A&E, in a climate of austerity, and its effects on the experiences and practices of healthcare professionals. It provides an analysis of the normative role of structures and regulations and explores how policy rules and functions impact on the perception and practice of professional duties and obligations in healthcare.

## Background

### Austerity Policies and the Drive for Efficiency in Healthcare

Since 2010, the UK has embarked on an ‘age of austerity’, characterised by budget cuts for welfare provisions, including social care [4, 5] and mental health [6]. Although most areas of public spending have seen their budgets reduced, the successive governments that imposed these austerity measures promised not to reduce the NHS budget [7]. The letter of this promise has been kept, but not necessarily its spirit [8]. According to the King’s Fund, the NHS budget increase is set at an average of 1.1% between 2010 and 2021, as opposed to an average increase of 4% that was the case since the foundation of the NHS in 1957 [9]. This below-the-expected-average increase means that healthcare services have been struggling to meet the population’s rising healthcare demands [10–12]. At the same time, the NHS is required to make £22 billion worth of efficiency savings [13], and achieve 2% yearly efficiency improvements by 2020 [14] to cover the increasing demand on its services.

Efficiency is understood as the most cost-effective way to use resources, including human resources, capital and equipment, in order to achieve certain outputs, such as number of patients treated and waiting times (intermediate outputs), or lives saved and life years gained (final outputs) [15]. Healthcare systems use a number of indicators to monitor efficiency [16]. One of the main indicators used is operational performance targets and protocols. The aim of these targets and protocols is to improve patient outcomes and reduce costs (final outputs) by reducing waiting times and streamlining processes (intermediate outputs). Meeting operational performance targets is seen as a main sign of providing quality care efficiently in the NHS [17]. As Palmer and Torgerson [15] caution, focusing on intermediate outputs as measure can lead to suboptimal final health outcomes and Smith notes that even though all indicators used have their limitations and weaknesses, when ‘properly used they can offer diagnostic information on where and why inefficiency is present’ [16].

For A&E Departments the most important indicator of optimum operational performance is the 4 h target. The 4 h target, which was first introduced in the early 2000s, stipulates that patients in A&E should be seen and either referred on or discharged within 4 h of admission. Initially the target was set at 100% but it was reduced to 98% in 2004 and then again to 95% in 2010, to allow for variation in patient needs. Yet, A&E Departments around the country have not been able to meet this target since July 2015, causing overcrowding and putting patient and staff safety at risk. In January 2017, the Secretary of Health announced that the 4 h target should be re-interpreted to only include ‘urgent’ cases [18], and in Government’s mandate to NHS England for 2018–19 put the target on hold with a plan to ‘deliver aggregate A&E performance in England at 95% within the course of 2019’ [19]. Other important operational targets have also been missed in recent years. For example, the referral-to-treatment for cancer has not been met for 3 years, and the referral-to-treatment for elective care has been missed since February 2016 [9].

In 2018, the Government announced a real terms increase for the NHS of £20.5 bn over 5 years [20]. While NHS Providers expressed hesitation concerning whether this funding would be enough to bring about the improvements required [21], the service committed towards striving for greater efficiency whilst providing ‘safe, high quality and compassionate care’ [22]. However, very little, if any information, has been given about how the balance between efficiency and compassionate care will be achieved [23]. The assumption seems to be that providing empathetic and compassionate care is ‘easy and cost-neutral’ [24].

Previous events suggest that the relationship between efficiency and empathy is not straightforward. The Mid-Staffordshire case in 2013 demonstrated that empathy and compassion can be side-lined when the main focus is on reaching operational targets and achieving financial balance. By pointing at issues such as overwork and lack of continuity, the Francis Report highlighted institutional and systemic structures that could impact on the provision of empathetic care, and which go beyond the emotional and cognitive ability of individuals to empathise [25]. Sir Robert Francis, who led the Mid Staffordshire inquiry, recently repeated his concerns about the ability to provide compassionate and empathetic care in the current austere and efficiency driven climate [26].

### Empathy and Compassion in Healthcare

Empathy signifies the ability to understand the world from someone else’s perspective, and requires the engagement of both cognitive and emotive abilities [27, 28]. The cognitive side of empathy denotes the ability to comprehend another person’s experiences, and the emotional component refers to the ability to join in another’s feelings. The empathetic engagement with another can lead to the desire to alleviate the other’s suffering, that is compassion [29]. Empathy, therefore, can be the precursor to compassion, as it can bring about the desire to help someone and address their needs. Conversely, compassion requires empathy, as it is through empathy that one understands another’s situation and predicament, and feels motivated to act [30, 31]. At this point, it would be useful to briefly introduce the concept of sympathy, only to distinguish it from those of empathy and compassion. Sympathy is ‘an instance of *feeling* another individual’s predicament …leading to emotional identification between the two individuals’ [32], rather than understanding of another’s situation that is empathy, or being motivated to help that is compassion. In recent years empathy has been critiqued [33–35]. Bloom [33], for example, argues that empathy can lead to immoral actions, and advocates for rational and distanced compassion instead. Persson and Savulescu [36] challenge Bloom’s thesis on the grounds that he wrongly defines empathy as *feeling* someone else’s emotions rather than *understanding* them, and on mistakenly juxtaposing reason (as a function of rationality) with empathy (as a function of intuition). They conclude that ‘reason is not an *alternative* to empathy: it needs empathy as a motivator, and empathy needs reason for its motivational force to be properly directed and encompassing’. What both Bloom and his critics, however, agree upon is that empathy motivates action, including compassionate action.

In healthcare, for the past few decades there has been a distinct and sustained movement away from a paternalistic model of care, where the healthcare professional dominates the decision-making process, towards a shared decision-making model that puts the patient in the centre of the therapeutic relationship [37–39]. In this patient-centred care model, empathy is fundamental [40]. Doctors and nurses are expected to not only provide competent treatment but also care, in an empathetic and compassionate way, to their patients. However, empathy does not come without costs. Listening and engaging with patients’ experiences and feelings requires time, and studies have shown that doctors who spent more time with patients are perceived by them as being more empathetic [41]. The emotional labour of empathy also requires material support. Time and resources are needed to allow healthcare professionals to process and reflect on their own feelings as a way of enhancing and maintain their ability to empathise [42]. Institutional structures, system priorities and the underlying ethical principles on which healthcare systems are built also impact on the healthcare professionals’ motivation and ability to perform their duties [43].

A number of studies have explored the impact of austerity on care provision [44–46] and on healthcare professionals themselves [47–49]. However, there has been no specific analysis of the relationship between efficiency and empathy in healthcare at times of austerity. This paper uses data collected as part of an empirical bioethics project on the effects of austerity on everyday experiences and professional duties [50]. It focuses on the practical and normative implications of the renewed drive for efficiency on the everyday practice of healthcare professionals, and on the professional and ethical character of the medical and nursing professions as a whole.

## Study and Methods

The data used in this paper were collected as part of a qualitative study, which set out to examine the everyday experiences of healthcare staff working in A&E Departments in England [50]. Between February and November 2017, 16 semi-structured interviews were conducted with nine doctors and seven nurses, all of different seniority, in three different A&E Departments in Central, South-East and North-West England. Two are teaching hospitals and one a regional hospital and the site locations covered rural and urban populations. Participants were recruited mainly via a general email sent to the A&E departments, with the help of the Clinical Lead or Research Nurse, to introduce the study. Posters were also put on notice boards and, on two occasions, the researchers were invited to attend departmental meetings to present the study and distribute information leaflets. Staff interested in participating emailed the researchers directly to arrange a convenient time for the interview. Fourteen interviewers were conducted face-to-face and two via skype, and lasted approximately 45 min (33–80 min). All interviews were recorded, transcribed verbatim, anonymised and analysed using thematic analysis. During the interviews the participants were asked to describe their experiences of working in A&E particularly since 20210–11, and to reflect on what they identified as professional and ethical issues. As is common practice in qualitative research, emerging themes in early interviews became the focus of questions in subsequent interviews. For example, early interviews suggested that empathy and efficiency were important concerns for these healthcare professionals, so we developed questions to pursue this line of inquiry in later interviews. Two researchers read the transcripts before coding them separately. Similarities and differences in the coding were discussed and the final analytic themes were developed collaboratively. This paper discusses the themes of efficiency, empathy and compassion as they emerged in the study. The study was approved by the Health Research Authority (HRA; IRAS 213721), and the Research and Development (R&D) offices of each hospital involved.

## Findings

### The Drive for Efficiency: Meeting Targets

The healthcare professionals interviewed acknowledged that their budget was not reduced in actual terms, but that cuts in services such as mental health and social care have had a significant knock-on effect on the A&E Departments. They described A&E as the ‘front face of the NHS’ and the ‘canary in the mine’, indicating that failings in the social and welfare system always manifest themselves at the A&E waiting rooms. In this climate of austerity, a major concern for these doctors and nurses is providing high quality care for an ever increasing number of patients whilst meeting the operational targets, particularly the 4 h target. In their view, the 4 h target has helped to improve patient care in so far as patients no longer have to wait for a long time to be seen. They also recognised that A&E Departments are not unfamiliar with dealing with high number of patients. For example, during the winter months A&E departments would normally expect an increase in admissions, followed by a return to normal numbers once the flu season is over. However, demand has remained at high levels throughout the year, putting pressure on staff who are still expected to meet the operational targets of the department. As Nurse 1 explains, the main reason why meeting targets is important in A&E is because these measures are directly linked to funding. If a hospital fails to achieve its targets, it is penalised by having its budget reduced:

Cause there is so much pressure to meet the targets and that’s what it comes down to. […] The risk of going into special measures, you lose a lot of funding and you get a lot of scrutiny on the Trust as a whole. (Nurse, 1)

Doctor 10 explained that the fear of missing targets and being penalised is so great that the hospital management appear to be interested in staffing numbers only in so far as this might affect their meeting targets:

So they (managers) are obsessed with the board (dashboard used to monitor the flow of patients), they are obsessed with how it’s run, they are obsessed. If you are doctors down, it matters to them, because it becomes more likely that you will have patients breaching. (Doctor, 10)

It is not only the 4 h target that has financial implications for a hospital Trust, the same approach is used for all targets and protocols, as a way of increasing compliance and achieving greater efficiency. Although doctors and nurses recognise that such protocols might be beneficial for individual patients, the effect they have on other patients, on the staff, and on the operation of the unit as a whole is not necessarily positive. Nurse 12 explained the implicit pressures on staff that are associated with a new sepsis protocol in their unit:

Which (the new sepsis protocol) is fantastic for the patient, of course it is, but the pressure that puts on the nurse to deliver that target is very hard. If you have got six septic patients coming in at once and seven other patients, it’s very hard, and this is where a lot of the stress comes from for nurses. […] Because it’s a target, then there is money attached to that target, the pressure from the management is very strong. (Nurse, 12)

### The Effects of the Drive for Efficiency

The introduction of targets and protocols might lead to improved health outcomes for the targeted patients but it creates problems for the management and care of patients whose conditions do not have financial incentives attached to them. The healthcare professionals’ disquiet with the increased “obsession with the board” stems from the fact that it makes their work-days more stressful and demanding. They are also concerned with what they perceive to be the (financial) motivation behind the urgency to meet targets. In their view, this urgency is not driven by a concern for patient care but, instead, by financial concerns:

There is an enormous pressure from management not to have patients in the emergency department for more than 4 h, and to make quick decisions, which is not always possible for people. People don’t follow prescriptions; they don’t follow formulas. […] I think to use the term bullying is very strong – but there is a culture of, certainly heavy pressure on staff to make decisions in a certain way to achieve a certain outcome, which is financially driven. (Doctor, 8)

In this highly constrained environment characterised by austerity of resources and “austerity of time” (Doctor, 9), healthcare professionals are required to make hard decisions, including deciding what they can offer to the patients and what will have to be left out. And, as Nurse 3 explains, it is often the caring side of medicine that suffers.

it is just rushing, rushing, rushing to do the best that you can. […] It doesn’t feel like I am giving the care that I would like to give.[…] I think what’s happening is we are choosing what to do because we can’t do it all. So we‘ll do our time critical medication and we’ll document but that then means that the care suffers. (Nurse, 3)

When it comes to efficiency indicators, tracking the time a patient spends in A&E is easy to measure and quantify. Yet, as one doctor explains, seeing someone quickly does not necessarily equate with providing good “holistic” care. In this professional’s view, the pressure for more efficiency makes them “less and less effective, so I think you feel… impotent is the right word, really.” (Doctor 2) This doctor goes on to explain that for those in positions of authority meeting the 4 h target and discharging more patients quickly is what A&E departments should be aiming at, but from the professional’s perspective the value is questionable: “From a government, NHS or even management point of view […] they can say […] “look we sent a lot of people home, that’s a good thing”. Well, how do you know it’s a good thing?” According to these professionals, what is easily measured, in this case the intermediate output of patient throughput, has replaced what ought to be the aim of efficiency, namely improved care.

The pressure to meet targets is leading departments to adopt models of organisation that resemble Fordist lines of production, rather than places of care. For example, interviewees explained that one nurse will be responsible for cannulating patients and taking bloods, and another will be administering drugs or checking electrocardiograms (ECGs). Even though this division of labour may help with the flow of patients through A&E, it also results in healthcare professionals becoming “de-skilled” (Nurse 12) as they become more specialised but less versatile.

### Efficiency Versus Caring for Patients: Effects on Empathy and Compassion

It is not only the practical skills that healthcare professionals lose in a task-oriented environment. They also lose their professional autonomy, namely the opportunity to exercise clinical judgement regarding the care of patients. According to Doctor 7 below, those in positions of power have a specific view of how medicine and care should be practiced, and they are using austerity and efficiency-driven arguments in order to alter the nature and ethos of healthcare. In the process of trying to change medicine from a “profession” into a “job” what is lost is not the clinical aspects of the role but the caring aspect of these professions:

To actually have people to properly look after people costs a lot more and they (the government) are not prepared to pay for it. … The quality of *caring*, you know, I‘ve seen that drop. (Doctor, 7)

It is interesting to witness an A&E doctor raising the issue of caring. The assumption is that A&E is a place where medical decisions need to be made quickly. And yet, as indicated here, appropriate healthcare cannot exclude the caring for patients. In a patient-centred model of care, empathy allows the healthcare professional to understand the patient’s perspective and provide appropriate, compassionate care. However, the opportunity to engage empathetically with a patient and demonstrate care and compassion can be lost in a underfunded and target-driven environment. For some, it is the enaction of empathy that is compassion that suffers in such an environment, as this nurse explains:

I think they [younger nurses] are very empathic, I think they are very caring, I think they are very hard working. […] they just work in an entirely different environment. […] it’s just they can’t do it. (Nurse, 12)

Yet for others, the operationalisation of medicine in the name of efficiency has more profound effects on the way they perceive themselves as well as the patients they treat, as Doctor 2 commented:

it is difficult to engage and empathise with patients. I think it kind of depersonalises them and you. It kind of makes you feel a bit less human […] as if you are a robot or a computer. (Doctor, 2)

## Discussion

Efficiency denotes the most cost-effective way to utilise healthcare resources, and is central to all healthcare systems, particularly to tax-funded ones like the NHS. Healthcare systems need to distribute human and material resources prudently, and this includes allocating doctors’ and nurses’ time and skills appropriate in order to maximise health benefits for patients. A number of performance indicators, such as the 4 h target and disease protocols, are used to monitor and measure efficiency [16]. By introducing targets and protocols the healthcare system ensures control over the way healthcare professionals’ use their time and deploy their expertise. Although many of these performance indicators were introduced before 2010, the recent period of austerity intensified the focus on, and urgency for efficiency. As A&E Departments saw admissions rise, meeting targets became increasing more difficult [51]. However, efforts to keep performance high meant that great pressure was placed on doctors and nurses, leading to stress and burnout [50]. The healthcare professionals interviewed for this study objected to what they perceived as an increased operationalisation of their professions. They argued that the “obsession with the board”, or electronic monitoring of patient throughput in A&E, resulted in a significant increase in their workload and stress; but more importantly, they objected to the way in which these targets and protocols curtailed their clinical judgement and hindered the practice of core professional values such as patient-centeredness, empathy and compassion. They felt that a system that was more concerned with processing patients rather than caring for them, removed the humanity from the therapeutic relationship and turned doctors and patients into “robots or a computer”.

### Performativity and the Changing (Moral) Character of Healthcare Professions

Ball’s performativity [52] can provide a useful lens through which to view and interpret these findings. According to Ball, performativity denotes ‘a technology, a culture and a mode of regulation that employs judgements, comparisons and displays as means of incentive, control, attrition and change –based on rewards and sanctions (both material and symbolic). The performances (of individual subjects or organizations) serve as measures of productivity or output, or displays of ‘quality’ or ‘moments’ of promotion or inspection. As such they stand for, encapsulate or represent the worth, quality or value of an individual or organization within a field of judgement’ [52]. By attaching financial incentives to targets and protocols, the healthcare system is indicating what is of significance and value and what is not. Efficiency’s normative force stems from its claim to better quality of care for all [15]. However, by conflating the final aim of improved care with outputs that are easy to measure and control, such as number of hours spent in A&E, the principle of efficiency becomes short-sighted and its normative force is questioned.

Measuring what is easily measured can be seen as a practical solution to the ‘formidable’ difficulties of ‘conceptualising, measuring and improving efficiency’ [16]. However, such practical solutions can have a profound impact on the health of patients but also on the moral space in which healthcare is practiced. The equation of efficiency with the meeting of targets and protocols, then, signifies not only what those in position of power consider to be important, but also what the purpose of healthcare should be. Through the use of targets and protocols, as a mode of incentivising and controlling behaviour, those responsible for structuring and managing the healthcare system, are also redefining the duties and obligations of doctors and nurses, and the core values of these professions. By attaching financial incentives on performance indicators, a new order of priorities is imposed on these professionals that circumvents or replaces their professional judgment. Doctors and nurses are pressured to process patients quickly rather than empathically engage with them. Thus ‘a new basis for ethical decision-making and moral judgement is erected’ [52] for the medical professions. In this system, a good doctor or nurse is one that follows instructions rather than one that uses their judgement and initiative; one that deals quickly with the physiological aspect of disease, rather than someone who considers patients holistically and engages with them empathetically.

The wider perception is that the ability to demonstrate empathy and act compassionately rests solely with the individual. This is why medical schools try to recruit compassionate and empathetic students [53, 54] and include empathy courses in the curriculum [55]. However, organisations cannot ensure empathy and compassion simply by hiring empathetic and compassionate employees [56]. The organisational structure, existing conditions and the ‘performances’ expected from employees, impact on peoples’ abilities, opportunity and resolve to exercise certain behaviours, such as empathy and compassion. Even when these behaviours are motivated by personal values or virtues, the social norms, systems and structures present in one’s setting can inhibit or facilitate their practice [57]. As Allmark notes, if the environment is hostile toward the expression of certain virtues, moral actors are less likely to develop and exercise them [58]. A healthcare structure that systematically promotes and rewards certain practices (e.g. seeing patients quickly) but does not allow space for others, such as empathy, implicitly guides and shapes the way right action is perceived by and for the healthcare professions. Crowding-out empathy and compassion from healthcare has the potential to fundamentally alter the moral orientation of these professions. From professions concerned with healing persons and relieving suffering [59], medicine and nursing would become more about fixing the ‘abnormalities of the structure and function of body organs and systems’ [60].

It was mentioned above that the majority of targets and protocols to which A&E doctors and nurses need to adhere were introduced years before the beginning of austerity in 2010. Austerity did not the cause the shift towards efficiency, but the ‘necessity’ of austerity and moral force of efficiency created the right conditions that facilitated changes in the healthcare professions. It is difficult to argue against calls for greater efficiency, particularly when the predominant rhetoric emphasises the necessity and inevitability of austerity. However, there is a growing consensus amongst economists and political theorists that austerity was driven more by political ideology rather than economic necessity [61–64]. Philip Alston, the UN special rapporteur, concluded that the austerity policies imposed on British people by the successive governments since 2010 brought about social re-engineering and fundamental changes in the values that underpin social systems and structures in the UK, including the healthcare system [3]. Efficiency is important for a publicly funded healthcare systems, but it should not be used as a way of crowding-out other essential values of healthcare provision, uncritically.

## Limitations of the Study

One of the major limitations of this study is the small number of interviews conducted, which makes the extrapolation and defence of general arguments regarding the effects of austerity difficult or even questionable. There might also be a risk of bias in our data, as it is possible that only those who felt strongly about the issue of austerity responded to the call for participation. There were practical reasons that prevented further data collection, relating to the length of time it took to get the appropriate ethics approvals and permissions from the local Research and Development officers of individual sites. However while increasing the number of interviews would have increased the amount of data available, preliminary analysis demonstrated that the study had reached data saturation, which aided the decision to stop data collection. Furthermore, the fact that the themes that emerged from the analysis corresponded with other studies and media reports on the topic of austerity gave the researchers further confidence about their value and validity.

## Conclusion

Recent austerity policies imposed by successive UK governments did not introduce the focus on efficiency in healthcare and its implementation through targets and protocols, but it intensified it. Arguments supporting austerity policies and the conditions generated by their application renewed impetus in healthcare towards efficiency-driven regulations, which pushed core healthcare values such as empathy to the periphery of the nursing and medical encounter. Healthcare professionals tasked with implementing these regulations perceived them as a way of curtailing their professional judgment and redefining their duties and obligations.

Policy technologies, to use Ball’s term, are not only responsible for redesigning the structure and form of healthcare systems, but also carry profound normative implications, which should not be ignored or overlooked. By using incentives and punishment, governments and healthcare systems control the way healthcare professionals perceive and enact their duties and obligations in practice. This way, these policies create the environment in which what counts as ‘right action’ is (re)defined.

The role of policies and institutional structures in setting the parameters of professional roles and in the functioning of professional values needs to be formally examined. Moving forward, a normative (re)evaluation of the role and practice of values such as empathy in the healthcare professions will be necessary, in light of the new context that is emerging in healthcare. It is important to extend the ethical discussion about values and virtues in the healthcare professions to include not only the individual practitioners but also the healthcare systems and social structures in which these practitioners operate.

## References

[1] Timms S (2010). Budget 2010: Securing the recovery.

[2] Living within our means speech (2008). UKPOL. http://www.ukpol.co.uk/david-cameron-2008-living-within-our-means-speech/. Accessed 15 June 2018.

[3] Alston P (2018). Statement on visit to the United Kingdom, by Professor Philip Alston.

[4] Hastings, A., Bailey, N., Bramley, G., Gannon, M., & Watkins, D. (2015). The cost of the cuts: The impact on local government and poorer communities. Joseph Rowntree Foundation.

[5] Hood A, Phillips D (2015). Benefit spending and reforms: The coalition governemnt’s record.

[6] McNicoll, A. (2015). Mental health trust funding down 8% from 2010 despite coalition’s drive for parity of esteem. Community Care.

[7] Spending Revivew 2010: George Osborne wields the axe. (2010, 20 October). *BBC News*.

[8] Appleby J, Baird B, Thomson J, Jabbal J (2015). The NHS under the coalition government. Part two: NHS performance.

[9] The King’s Fund (2017). Does the NHS need more money?: The King’s Fund, London.

[10] Murray R, Jabbal J, Thomson J, Baird B, Maguire D (2017). Quarterly monitoring report: June 2017.

[11] Robertson R, Wenzel L, Thomspon J, Charles A (2017). Understadning NHS financial pressures: How are they affecting patient care?.

[12] Maybin R, Charles A, Honeyman M (2016). Understanding quality in district nurisng services: Learning from patients, carers and staff.

[13] Department of Health and Social Care (2010). 2010 to 2015 government policy: NHS efficiency. In: D. O. H. A. S. Care (Ed.).

[14] NHS Improvement (2016). 2020 Objectives. NHS Improvement.

[15] Palmer S, Torgerson DJ (1999). Definitions of efficiency. BMJ.

[16] Smith PC (2012). What is the scope for health system efficiency gains and how can they be achieved. Eurohealth.

[17] NHS Providers (2017). Mission Impossible? The task for NHS Providers in 2017/18. NHS Providers.

[18] Jeremy Hunt ditches four-hour target as A&E crisis deepens. (2017, 9 January). *The Guardian*.

[19] Department of Health and Social Care (2018). The Government’s mandate to NHS England for 2018–19.

[20] HM Treasury (2018). Budget 2018. HM Treasury.

[21] NHS Providers (2018). Commitments to mental health sends right message but more support for core services needed.

[22] NHS Improvement (2018). Annual report. NHS Improvement.

[23] NHS Providers (2018). Making the most of the money: Efficiency and the long-term plan. NHS Providers.

[24] Bivins R, Tierney S, Seers K (2017). Compassionate care: Not easy, not free, not only nurses. BMJ Quality & Safety.

[25] Francis R (2013). Report of the mid staffordshire NHS foundation trust public inquiry.

[26] Government says NHS hospitals can wring out another £300 m in efficiency savings. (2017, 8 November). *Independent*.

[27] Hojat M, Gonnella JS, Nasca TJ, Mangione S, Vergare M, Magee M (2002). Physician empathy: Definition, components, measurement, and relationship to gender and specialty. American Journal of Psychiatry.

[28] Hojat M, Mangione S, Nasca TJ, Cohen MJ, Gonnella JS, Erdmann JB (2001). The Jefferson scale of physician empathy: Development and preliminary psychometric data. Educational and Psychological Measurement.

[29] Singer T, Klimecki OM (2014). Empathy and compassion. Current Biology.

[30] Nussbaum M (1996). Compassion: The basic social emotion. Social Philosophy and Policy.

[31] Goetz JL, Keltner D, Simon-Thomas E (2010). Compassion: An evolutionary analysis and empirical review. Psychological Bulletin.

[32] Switankowsky I (2000). Sympathy and empathy. Philosophy Today.

[33] Bloom P (2016). Against empathy: The case for rational compassion.

[34] Prinz J, Coplan A, Goldie P (2011). Is empathy necessary for morality?. Empathy.

[35] Prinz J (2011). Against empathy. Southern Journal of Philosophy.

[36] Persson I, Savulescu J (2018). The moral importance of reflective empathy. Neuroethics.

[37] Emanuel EJ, Emanuel LL (1992). Four models of the physician-patient relationship. JAMA.

[38] Frosch DL, Kaplan RM (1999). Shared decision making in clinical medicine: Past research and future directions. American Journal of Preventive Medicine.

[39] Chin JJ (2002). Doctor-patient relationship: From medical paternalism to enhanced autonomy. Singapore Medical Journal.

[40] Bauchat JR, Seropian M, Jeffries PR (2016). Communication and empathy in the patient-centered care model—why simulation-based training is not optional. Clinical Simulation In Nursing.

[41] Neumann M, Bensing J, Wirtz M, Wübker A, Scheffer C, Tauschel D (2011). The impact of financial incentives on physician empathy: A study from the perspective of patients with private and statutory health insurance. Patient Education and Counseling.

[42] Kerasidou A, Horn R (2016). Making space for empathy: Supporting doctors in the emotional labour of clinical care. BMC Medical Ethics.

[43] Kerasidou, A., & Horn, R. (2018). Empathy in healthcare: The limits and scope of empathy in public and private systems. In: T. Feiler, J. Hordern, & A. Papanikitas (Eds.), *Marketisation, ethics and healthcare: policy, practice and moral formation* (pp. 163–173, Key Themes in Health and Society). London: Routledge.

[44] Cummins I (2018). The impact of austerity on mental health service provision: A UK perspective. International Journal of Environmental Research and Public Health.

[45] Roberts, A., Marshall, L., & Charlesworth, A. (2012). A decade of austerity? The funding pressures facing the NHS from 2010/11 to 2021/22. In: N. Trust (Ed.). London: Nuffield Trust.

[46] Janssen D, Jongen W, Schröder-Bäck P (2016). Exploring the impact of austerity-driven policy reforms on the quality of the long-term care provision for older people in Belgium and the Netherlands. Journal of Aging Studies.

[47] Kerasidou A, Kingori P, Legido-Quigley H (2016). You have to keep fighting: Maintaining healthcare services and professionalism on the frontline of austerity in Greece. International Journal for Equity in Health.

[48] Russo G, Pires CA, Perelman J, Gonçalves L, Barros PP (2017). Exploring public sector physicians’ resilience, reactions and coping strategies in times of economic crisis; findings from a survey in Portugal’s capital city area. BMC Health Services Research.

[49] Humphries N, McAleese S, Matthews A, Brugha R (2015). Emigration is a matter of self-preservation. The working conditions are killing us slowly: Qualitative insights into health professional emigration from Ireland. Human Resources for Health.

[50] Kerasidou A, Kingori P (2019). Austerity measures and the transforming role of A&E professionals in a weakening welfare system. PLoS ONE.

[51] The King’s Fund (2017). What’s going on with A&E waiting times?: The King’s Fund, London.

[52] Ball SJ (2003). The teacher’s soul and the terrors of performativity. Journal of Education Policy.

[53] Lancaster University Medical School (2019). What else are we looking for? https://www.lancaster.ac.uk/lms/study-with-us/undergraduate/mbchb/entry-requirements-and-selection-process/what-else-are-we-looking-for/. Accessed 23 January 2019.

[54] The complete university guide (2019). Medicine applications: Multiple mini interviews (MMI). https://www.thecompleteuniversityguide.co.uk/courses/medicine/applying-to-medicine-multiple-mini-interviews-(mmis)/. Accessed 23 January 2019.

[55] Boker JR, Shapiro J, Morrison E (2004). Teaching empathy to first year medical students: Evaluation of an elective literature and medicine course. Education for Health.

[56] Dutton JE, Worline MC, Frost PJ, Lilius J (2006). Explaining compassion organizing. Administrative Science Quarterly.

[57] Buchanan A (2002). Social moral epistemology. Social Philosophy and Policy.

[58] Allmark P (2013). Virtue and austerity. Nursing Philosophy.

[59] Cassel EJ (1982). The nature of suffering and the goals of medicine. New England Journal of Medicine.

[60] Eisenberg L (1977). Disease and illness. Distinctions between professional and popular ideas of sickness. Culture, Medicine and Psychiatry.

[61] Stiglitz, J. E. (2014). Europe’s austerity zombies. *Project Syndicate*.

[62] Blyth M (2013). Austerity: The history of a dangerous idea.

[63] Austerity policies do more harm than good, IMF study concludes. (2016, 27 May). *The Guardian*.

[64] Emmerson C (2017). Two parliaments of pain: The UK public finances 2010 to 2017.

